# Recommendations for initiation and cessation of enzyme replacement therapy in patients with Fabry disease: the European Fabry Working Group consensus document

**DOI:** 10.1186/s13023-015-0253-6

**Published:** 2015-03-27

**Authors:** Marieke Biegstraaten, Reynir Arngrímsson, Frederic Barbey, Lut Boks, Franco Cecchi, Patrick B Deegan, Ulla Feldt-Rasmussen, Tarekegn Geberhiwot, Dominique P Germain, Chris Hendriksz, Derralynn A Hughes, Ilkka Kantola, Nesrin Karabul, Christine Lavery, Gabor E Linthorst, Atul Mehta, Erica van de Mheen, João P Oliveira, Rossella Parini, Uma Ramaswami, Michael Rudnicki, Andreas Serra, Claudia Sommer, Gere Sunder-Plassmann, Einar Svarstad, Annelies Sweeb, Wim Terryn, Anna Tylki-Szymanska, Camilla Tøndel, Bojan Vujkovac, Frank Weidemann, Frits A Wijburg, Peter Woolfson, Carla EM Hollak

**Affiliations:** Department of Internal Medicine, Division Endocrinology and Metabolism, Academic Medical Center, PO Box 22660, Amsterdam, 1100 DD The Netherlands; Biomedical Center, University of Iceland and Landspitali University Hospital, Reykjavík, Iceland; Center of Molecular Diseases, Centre Hospitalier Universitaire Vaudois, Lausanne, Switzerland; Fabry International Network (FIN), Amersham, UK; Department of Clinical and Experimental Medicine, University of Florence, Florence, Italy; Department of Medicine, Addenbrooke’s Hospital and University of Cambridge, Cambridge, UK; Department of Medical Endocrinology, Copenhagen University Hospital, Copenhagen, Denmark; Department of Endocrinology, University Hospitals Birmingham NHS Foundation Trust, Birmingham, UK; Division of Medical Genetics, University of Versailles, Montigny, France; Department of Adult Inherited Metabolic Disorders, Manchester Academic Health Science Centre, Manchester, UK; Department of Haematology, Lysosomal Storage Disorders Unit, Royal Free Hospital, University College London, London, UK; Division of Medicine, Turku University Hospital, Turku, Finland; Villa Metabolica, Centre for Paediatric and Adolescent Medicine, Mainz, Germany; Fabry Support and Information Group the Netherlands (FSIGN), Oosterwolde, the Netherlands; Department of Genetics, University of Porto & São João Hospital Centre, Porto, Portugal; Rare Metabolic Diseases Unit, Paediatric Clinic, San Gerardo University Hospital, Monza, Italy; Lysosomal Disorders Unit, Institute of Immunity and Transplantation, Royal Free Hospital, London, UK; Department of Internal Medicine IV, Division Nephrology and Hypertension, Medical University Innsbruck, Innsbruck, Austria; Epidemiology, Biostatistics and Prevention Institute, University of Zurich, Zurich, Switzerland; Department of Neurology, University of Würzburg, Würzburg, Germany; Department of Medicine III, Division Nephrology and Dialysis, Medical University of Vienna, Vienna, Austria; Department of Medicine, Haukeland University Hospital and Department of Clinical Medicine, University of Bergen, Bergen, Norway; Department of Internal Medicine, Division of Nephrology, Ghent University Hospital, Ghent, Belgium; Department of Paediatrics, Nutrition and Metabolic Diseases, The Children’s Memorial Health Institute, Warsaw, Poland; Clinical Trial Unit/Department of Paediatrics, Haukeland University Hospital, Bergen, Norway; General Hospital Slovenj Gradec, Slovenj Gradec, Slovenia; Innere Klinik II, Katharinen Hospital Unna, Unna, Germany; Department of Paediatrics, Academic Medical Center, Amsterdam, The Netherlands; Department of Cardiology, Salford Royal Hospital NHS Foundation Trust, Manchester, UK

**Keywords:** Fabry disease, Enzyme replacement therapy, Recommendations, Delphi procedure

## Abstract

**Introduction:**

Fabry disease (FD) is a lysosomal storage disorder resulting in progressive nervous system, kidney and heart disease. Enzyme replacement therapy (ERT) may halt or attenuate disease progression. Since administration is burdensome and expensive, appropriate use is mandatory. We aimed to define European consensus recommendations for the initiation and cessation of ERT in patients with FD.

**Methods:**

A Delphi procedure was conducted with an online survey (n = 28) and a meeting (n = 15). Patient organization representatives were present at the meeting to give their views. Recommendations were accepted with ≥75% agreement and no disagreement.

**Results:**

For classically affected males, consensus was achieved that ERT is recommended as soon as there are early clinical signs of kidney, heart or brain involvement, but may be considered in patients of ≥16 years in the absence of clinical signs or symptoms of organ involvement. Classically affected females and males with non-classical FD should be treated as soon as there are early clinical signs of kidney, heart or brain involvement, while treatment may be considered in females with non-classical FD with early clinical signs that are considered to be due to FD. Consensus was achieved that treatment should not be withheld from patients with severe renal insufficiency (GFR < 45 ml/min/1.73 m^2^) and from those on dialysis or with cognitive decline, but carefully considered on an individual basis. Stopping ERT may be considered in patients with end stage FD or other co-morbidities, leading to a life expectancy of <1 year. In those with cognitive decline of any cause, or lack of response for 1 year when the sole indication for ERT is neuropathic pain, stopping ERT may be considered. Also, in patients with end stage renal disease, without an option for renal transplantation, in combination with advanced heart failure (NYHA class IV), cessation of ERT should be considered. ERT in patients who are non-compliant or fail to attend regularly at visits should be stopped.

**Conclusion:**

The recommendations can be used as a benchmark for initiation and cessation of ERT, although final decisions should be made on an individual basis. Future collaborative efforts are needed for optimization of these recommendations.

**Electronic supplementary material:**

The online version of this article (doi:10.1186/s13023-015-0253-6) contains supplementary material, which is available to authorized users.

## Background

Diagnostic criteria and treatment options are often a matter of debate and controversy, especially in the field of rare diseases, such as Fabry disease (FD). The European Fabry Working Group (EFWG) is an independent organization of primarily physicians involved in the treatment of FD throughout Europe. In this paper we report the results generated by EFWG physicians, with additional comments from patient representatives, on consensus recommendations for initiation and cessation of enzyme replacement therapy (ERT) in FD.

### Fabry disease

FD (McKusick 301500) is an X-linked multisystemic lysosomal storage disorder with an estimated birth prevalence of 1:40.000-170.000 [1-3]. Late onset forms of the disease are more frequent [4]. The disease is caused by a deficiency of the lysosomal hydrolase α-galactosidase A (AGAL-A), resulting in storage of primarily globotriaosylceramide (Gb3) [5,6]. More than 600 variants in the α-galactosidase A (GLA) gene have been described, most of which appear in single families [1]. In hemizygous males, the disease may present itself during childhood or adolescence with characteristic features such as neuropathic pain, hypo- or anhidrosis, disseminated angiokeratoma, cornea verticillata, and microalbuminuria. At a later age, progressive kidney disease, hypertrophic cardiomyopathy and cerebrovascular disease (including stroke) can occur. This disease phenotype is currently reported as classical FD. Heterozygous females may also be affected and generally demonstrate a more variable and attenuated phenotype. In both males and females, life expectancy is diminished, although this is more apparent in males [7,8].

Substantial intrafamilial and interfamilial variability in age of disease onset and disease progression exists [9]. In the past five years, evidence has emerged that some male patients with a proven disease causing GLA gene mutation and reduced AGAL-A activity do not express the complete set of signs and symptoms as they occur in the cases with classical FD [1]. This phenotype may be referred to as non-classical FD. Patients with non-classical FD may present with one single non-specific symptom such as chronic kidney disease or left ventricular hypertrophy (LVH). These patients as well as female patients contribute significantly to the phenotypical heterogeneity of FD. On the other hand, the misclassification of some patients with a genetic variant of unknown significance (GVUS) as Fabry disease is an additional challenge [4] (see also Table 1), which may result in inappropriate initiation of enzyme treatment.

**Table 1:** ***Diagnostic criteria for a definite diagnosis of FD*** **(adopted from Smid et al. with permission** [24]**)**

**Definite diagnosis of FD**	
**Males**	**Females**
GLA mutation	GLA mutation
+	+
AGAL-A deficiency of ≤5% of mean reference value in leukocytes	normal or deficient AGAL-A in leukocytes
+	+
**A or B or C**	
A	
≥1 characteristic FD sign/symptom (Fabry neuropathic pain, cornea verticillata or clustered angiokeratoma)*	
B	
an increase of plasma (lyso)Gb3 (within range of males with definite FD diagnosis)	
C	
a family member with a definite FD diagnosis carrying the same GLA mutation	
**Uncertain diagnosis of FD**	
**Males/Females**	
All patients presenting with a non-specific FD sign (such as LVH, stroke at young age, proteinuria) who do not fulfil the criteria for a definite diagnosis of FD have a GLA GVUS. Further evaluations are needed, following diagnostic algorithms**.	
**Gold standard for uncertain FD diagnoses**	
In subjects with an uncertain FD diagnosis, a GVUS and a non-specific FD sign, the demonstration of characteristic storage in the affected organ (e.g. heart, kidney, aside from skin) by electron microscopy analysis, according to the judgment of an expert pathologist, in the absence of medication that can lead to storage, confirms FD.	

*Definitions:

Fabry neuropathic pain meets the ‘characteristic clinical criteria’ if there is neuropathic pain in hands and/or feet, starting before age 18 years or increasing with heat, fever. Quantitative sensory testing (QST) reveals a decreased cold detection threshold and the intraepidermal nerve fiber density is increased. There is no other cause for neuropathic pain.

Angiokeratoma meet the ‘characteristic clinical criteria’ if they are clustered and present in characteristic areas: bathing trunk area, lips, and umbilicus. There is no other cause for angiokeratoma.

Cornea verticillata meets the ‘characteristic clinical criteria’ if there is a whorl like pattern of corneal opacities. There is no other cause (medication induced, among other: Amiodarone, Chloroquine).

**For organ specific algorithms see Smid et al. [24] and Van der Tol et al. [41].

GLA mutation = mutation in the α-galactosidase A gene; AGAL-A = the lysosomal hydrolase α-galactosidase A; FD = Fabry disease; Gb3 = globotriaosylceramide; LVH = left ventricular hypertrophy; GVUS = genetic variant of unknown significance.

ERT with either agalsidase alfa or agasidase beta has been developed for FD treatment. In 2001, both agalsidases were authorized by the European Medicines Agency “*Under Exceptional Circumstances*” which means that it was deemed impossible for the applicant to provide comprehensive data on the efficacy and safety of the drug, since FD is very rare. Agalsidase alfa is authorized at a dose of 0.2 mg/kg (Shire, Lexington, MA, USA) and agalsidase beta at a dose of 0.3 - 1.0 mg/kg (Genzyme, a Sanofi company, Cambridge, MA, USA). No studies to date have shown convincing evidence on clinical grounds for superiority or non-inferiority of either one of these enzymes in head to head comparative studies [10,11]. In general, ERT may decrease cardiac mass [12-15] and reduce renal Gb3-accumulations [16-18], while the effects on nervous system disease and renal function are less well established [15,19-22]. In addition, effects of treatment in patients with non-classical FD are in general not studied separately and obscure evaluation of outcomes. More recent studies have focused on specific features, such as cardiac fibrosis or severe renal dysfunction, which may compromise effectiveness of ERT, since the presence of these features points to irreversible organ damage [17,19,23]. Based upon these insights, early initiation of therapy is frequently advocated. However, “early initiation of therapy” has not been defined until now. In addition, it may be questioned whether patients with end stage disease or co-morbidities may still benefit from treatment. Several local and national guidelines and protocols exist with criteria to start ERT, some also defined stopping criteria for ERT, but so far, no international consensus has been obtained.

## Methods

### Patients

A diagnosis of FD can be challenging in individuals with a GLA mutation in the absence of characteristic phenotypic or biochemical FD features. For the purpose of this consensus procedure, only patients with a definite diagnosis of FD were discussed. In a separate study, diagnostic criteria have been addressed, see Table 1 [24].

### Consensus procedure

Physicians and researchers involved in daily care for FD (n = 41) were invited to participate in the consensus procedure and were asked to send local or national documents with information on start and stop criteria used in their country. Participating experts (n = 28) were asked to disclose any conflicts of interests in writing (see competing interests). A modified Delphi technique was used to develop group consensus on start and stop criteria for ERT in FD. For the first round, a background document was compiled by the study team (MB, CHo) with information on national and local treatment protocols, an overview of inclusion and exclusion criteria applied in randomised controlled trials, and a summary of studies that addressed the issue of limited effectiveness of ERT in patients with renal or cardiac features potentially influencing the outcome of ERT (for results of literature review, see Additional file 1). Also, studies on the effects of ERT on the brain (TIA/strokes, white matter lesions) [12,19-23] and pain [25,26], and studies on the effectiveness of ERT in children [27-32] were included, as well as two studies on effects of antibodies [33,34]. Subsequently, an online questionnaire was set up by the study team to discuss initiation and cessation criteria. The panellists were asked to assess criteria on a 5-point Likert scale (1 = strongly disagree, 2 = disagree, 3 = neither agree nor disagree, 4 = agree, 5 = strongly agree) and were invited to add comments and suggestions. This survey was followed by a face-to-face meeting with presentation and discussion of anonymized results (absolute scores and comments). Patient representatives from the Fabry International Network (FIN) and the Fabry Support and Information Group from the Netherlands (FSIGN) were invited to participate in the discussions. After discussion statements were modified if required, and panellists voted again using the 5-point Likert scale. Final recommendations were refined via a draft manuscript on which panellists were asked to comment by email.

### Statistical considerations

A criterion is used in the treatment recommendations only if there was at least 75% agreement and no disagreement (i.e. ≥75% of the panellists voted 1 or 2 while none voted 4 or 5, and vice versa).

## Results

### Consensus panel

A list of physicians and researchers involved in daily care for FD was prepared. This list consisted of 41 experts who had expressed their interest in joining the EFWG or who were known for their contributions to the field. Twenty-eight (68%) responded to the online survey and 15 (37%) participated at the face-to-face meeting. Four patient representatives from the FIN and FSIGN (CL, LB, ASw, EM) attended the meeting and participated in the discussions. All experts and patient representatives gave their feedback on the draft manuscript, which was subsequently amended according to their comments. All experts finally agreed with the results presented in this report.

### Classes of recommendation

During the face-to-face meeting it was agreed to grade the strength of recommendations according to the scale as outlined in Table 2 [35] and statements were modified according to this scale. It should be emphasized here that the grading was primarily based on clinical experience, observational studies and a limited number of randomised controlled trials that used mostly surrogate endpoints.

**Table 2:** **Classes of recommendation**

**Class I**	Evidence and/or general agreement that a given treatment or procedure is beneficial, useful, effective	Is recommended/is indicated
**Class II**	Conflicting evidence and/or a divergence of opinion about the usefulness/efficacy of the given treatment or procedure	
***Class IIA***	Weight of evidence/opinion is in favour of usefulness/efficacy	Should be considered
***Class IIB***	usefulness/efficacy is less well established by evidence/opinion	May be considered
**Class III**	Evidence or general agreement that the given treatment or procedure is not useful/effective, and in some cases may be harmful	Is not recommended

### Overall consensus

The online survey consisted of 57 statements. Thirty-four (60%) of these 57 statements were discussed and modified during the face-to face meeting, and one statement was added, making a total of 58 statements of which 35 (60%) were discussed during the meeting. A statement was first formulated as a class I recommendation (i.e. “treatment with ERT is recommended” or “it is recommended to stop treatment with ERT”) followed by lower classes of recommendation (Classes IIA and IIB) if there was no consensus. If no consensus was achieved for a statement even when it was formulated as a Class IIB recommendation (i.e. “treatment with ERT may be considered” or “it may be considered to stop treatment with ERT”), it was concluded that no consensus could be achieved for that specific statement. Consensus was achieved for 30 (86%) of these 35 statements (see Additional file 2: Table S1 and Additional file 3: Table S2). Six (10%) of the remaining 23 statements were omitted as the group considered these statements irrelevant, and 17 (29%) were either considered in the context of one of the other statements or not discussed during the face-to-face meeting due to time constraints.

### Consensus criteria for initiation of ERT

The group agreed that a differentiation should be made between male and female patients, and between patients with classical and non-classical FD. The division of females into classical and non-classical FD is based on the presence or absence of clustered angiokeratoma, cornea verticillata, or a very high (lyso)Gb3 level. For males with classical FD, consensus was achieved that treatment with ERT may be considered in patients of 16 years or older even if they have no symptoms or clinical signs of organ involvement (Class IIB recommendation). The diagnosis of classical FD in these patients is based on the presence of a GLA mutation, absent or very low residual enzyme activity, and the presence of at least one of the following: angiokeratoma, cornea verticillata, or a very high (lyso)Gb3 level. Classically affected males and females and males with non-classical FD should be treated as soon as there are early signs of organ involvement (kidney, heart and/or CNS signs) consistent with FD and not fully explained by other pathology (Class I recommendation), while treatment may be considered in females with non-classical FD and early clinical signs consistent with FD (Class IIB recommendation). Organ specific criteria for treatment initiation are depicted in Table 3.

**Table 3:** **Consensus criteria for initiation of ERT**

	**No signs or symptoms**	Renal*	Cardiac*	CNS*	Pain*	GI*
**Classical FD, males**	if ≥ 16 years of age (Class IIB)	- microalbuminuria^†^ (Class I)	- cardiac hypertrophy (MWT > 12 mm) without (or only minimal signs of) fibrosis (Class I)	- WMLs (Class IIB)	- neuropathic pain (Class IIA)	GI symptoms (Class IIA if < 16 years of age, Class IIB if > 16 years of age)
		- proteinuria^†^ (Class I)		- TIA/stroke (Class IIA)		
		- renal insufficiency (GFR 60–90)^#^ (Class I)	- signs of cardiac rhythm disturbances^$^ (Class I)	- hearing loss, corrected for age (Class IIB)	- neuropathic pain even if completely controlled (not interfering with daily activities) with pain medication (Class IIB)	
		- renal insufficiency (GFR 45–60)^#^ (Class IIB)				
**Non-classical FD, males**		- microalbuminuria^†^ (Class I)	- cardiac hypertrophy (MWT > 12 mm) without (or only minimal signs of) fibrosis (Class I)	- WMLs (Class IIB)	- neuropathic pain (Class IIA)	GI symptoms (Class IIA if < 16 years of age, Class
		- proteinuria^†^ (Class I)		- TIA/stroke (Class IIA)	- neuropathic pain even if completely controlled (not interfering with daily activities) with pain medication (Class IIB)	
		- renal insufficiency (GFR 60–90)^#^ (Class IIA)	- signs of cardiac rhythm disturbances^$^ (Class I)	- hearing loss, corrected for age (Class IIB)		IIB if > 16 years of age)
		- renal insufficiency (GFR 45–60)^#^ (Class IIB)				
**Classical FD, females**		- microalbuminuria^†^ (Class IIB)	- cardiac hypertrophy (MWT > 12 mm) without (or only minimal signs of) fibrosis (Class I)	- WMLs (Class IIB)	- neuropathic pain (Class IIA)	GI symptoms (Class IIA if < 16 years of age, Class IIB if > 16 years of age)
		- proteinuria^†^ (Class IIB)		- TIA/stroke (Class IIA)	- neuropathic pain even if completely controlled (not interfering with daily activities) with pain medication (Class IIB)	
		- renal insufficiency (GFR 60–90)^#^ (Class IIA)		- hearing loss, corrected for age (Class IIB)		
		- renal insufficiency (GFR 45–60)^#^ (Class IIB)	- signs of cardiac rhythm disturbances^$^ (Class I)			
**Non-classical FD, females**		- microalbuminuria^†^ (Class IIB)	- cardiac hypertrophy (MWT > 12 mm) without (or only minimal signs of) fibrosis (Class I)	- WMLs (Class IIB)	- neuropathic pain (Class IIA)	GI symptoms (Class IIA if < 16 years of age, Class IIB if > 16 years of age)
		- proteinuria† (Class IIB)		- TIA/stroke (Class IIA)	- neuropathic pain even if completely controlled (not interfering with daily activities) with pain medication (Class IIB)	
		- renal insufficiency (GFR 60–90)^#^ (Class IIB)		- hearing loss, corrected for age (Class IIB)		
		- renal insufficiency (GFR 45–60)^#^ (Class IIB)	- signs of cardiac rhythm disturbances^$^ (Class I)			

*consistent with FD and not fully explained by other pathology; ^†^according to international guidelines of kidney disease, KDIGO criteria; ^#^in ml/min/1.73 m^2^ corrected for age (>40 years: −1 ml/min/1.73 m^2^/year); ^$^sinus bradycardia, AF, repolarization disorders; ERT = enzyme replacement therapy; GFR = glomerular filtration rate; MWT = maximal wall thickness; CNS = central nervous system; WMLs = white matter lesions; TIA = transient ischemic attack; GI = gastrointestinal.

The statement on hyperfiltration (GFR > 130 ml/min/1.73 m^2^ corrected for age (>40 years: −1 ml/min/1.73 m^2^/year [36])) as a reason to start ERT was omitted because the clinical relevance of this finding was considered unclear. Panellists agreed that treatment should not be withheld in patients with severe renal insufficiency (GFR < 45 ml/min/1.73 m^2^ corrected for age (>40 years: −1 ml/min/1.73 m^2^/year)). Likewise, it was agreed that treatment should not be withheld in patients on dialysis even if they are not eligible for a renal transplant, and in patients with cognitive decline of any cause. Initiation or continuation treatment with ERT in these patients should be carefully considered on an individual basis. It was emphasised that consent to treatment is needed, preferably from the patient (Class I recommendations).

### Consensus criteria to stop or not start ERT

Criteria to stop or not start ERT are summarized in Table 4. These criteria apply to both male and female patients with classical and non-classical disease except for the criterion ‘lack of response when the sole indication for ERT is neuropathic pain’ which applies to all but male patients with classical FD. This exception was made since males with classical FD and neuropathic pain are considered to be at high risk for developing clinical signs of organ involvement within a short period of time.

**Table 4:** **Consensus criteria to stop or not start ERT**

**Stop criteria**	**Class of recommendation**
Non-compliance > 50% of infusions	Class I
Failure to attend regularly (according to local guidelines) at FU visits	Class I
Persistent life threatening or severe infusion reactions that do not respond to prophylaxis, e.g. anaphylaxis	Class I
Patient request	Class I
End stage renal disease, without an option for renal transplantation, in combination with advanced heart failure (NYHA class IV)	Class IIA
End stage FD or other comorbidities with a life expectancy of < 1 year	Class IIB
Severe cognitive decline of any cause	Class IIB
Lack of response for 1 year when the sole indication for ERT is neuropathic pain while receiving maximum supportive care*	Class IIB
**Criteria for not starting ERT**	**Class of recommendation**
Advanced cardiac disease with extensive fibrosis [37] if cardiac disease is the sole treatment indication^†^	Class I
End stage renal disease, without an option for renal transplantation, in combination with advanced heart failure (NYHA class IV)	Class IIA
End stage FD or other comorbidities with a life expectancy of < 1 year	Class IIB
Severe cognitive decline of any cause	Class IIB

*does not apply to male patients with the classical phenotype; ^†^consistent with FD and not fully explained by other pathology; ERT = enzyme replacement therapy; FD = Fabry disease; FU = follow up; NYHA = New York Heart Association.

Treatment with ERT is not recommended for solely cardiac indications (i.e. there are no other signs or symptoms for which treatment may be recommended) in FD patients with advanced cardiac disease with extensive fibrosis [37] consistent with FD and not fully explained by other pathology.

### Statements for which no consensus was achieved

Additional file 4: Table S3 lists the statements for which no consensus was achieved. The timing of initiation of ERT in young males with classical FD without any symptoms or clinical signs of organ involvement was extensively discussed among panellists. Although it was agreed that treatment of these patients should not be initiated at the age of 0–5 years, and that treatment may be considered in patients of 16 years or older, some advocated for initiation of treatment at the age of 6–10 years (33%) or 11–15 years (47%).

There was no consensus on the statement that treatment with ERT is recommended in patients with FD with early signs of cardiac disease (unspecified), while the panel agreed on more specific cardiac abnormalities as criteria for initiation of ERT (see Table 3). In addition, it was discussed whether podocyte storage as shown on kidney biopsy as only sign of organ involvement would be sufficient to start treatment with ERT. Two-third disagreed while one-third neither agreed nor disagreed.

Panellists did not agree on the statement that treatment with ERT is not recommended for renal indication in male patients with classical FD who have renal insufficiency (GFR < 45 ml/min/1.73 m^2^ corrected for age (>40 years: −1 ml/min/1.73 m^2^/year)). Forty percent agreed with this statement, while 20% disagreed. The remaining 40% neither agreed nor disagreed. Most panellists (87%) agreed that treatment with ERT may be considered in patients with severe renal insufficiency (GFR < 45 ml/min/1.73 m^2^ corrected for age (>40 years: −1 ml/min/1.73 m^2^/year)). However, 1 panellist felt that renal function will deteriorate anyway in these patients, and therefore no consensus was achieved.

### Specific items brought forward by patient representatives

Patient representatives expressed their concerns about the statement to consider stopping treatment with ERT in patients with FD and recurrent strokes. They emphasised that these patients might still have good quality of life and benefit from treatment in other ways. It was therefore decided to omit this statement. A similar consideration applies to patients with cognitive decline. After discussion, consensus was achieved that treatment should not be withheld in patients with cognitive decline of any cause (including recurrent strokes) while it was emphasised that consent to treatment is needed, preferably from the patient (Class I recommendation). In addition, it was agreed that stopping treatment may be considered in patients with severe cognitive decline (Class IIB recommendation) but it was emphasised that cessation of ERT in these patients should be carefully considered on an individual basis.

It was endorsed that treatment with ERT should be stopped in patients who are non-compliant (missing >50% of their infusions) or fail to attend regularly at visits (Class I recommendations).

## Discussion

Several national and local protocols exist with different sets of criteria for the initiation and/or cessation of ERT in FD. This divergence is mainly caused by the fact that currently available evidence is limited. With the use of an adapted Delphi procedure, a consensus panel consisting of European FD experts deliberated the recommendations for the initiation and cessation of ERT, reflecting the current standard of evidence and practice. Comments and suggestions from FD patient organization representatives were also included.

We emphasise that our recommendations are only applicable to patients with a definite diagnosis of FD according to definitions provided in Table 1. This is of the utmost importance to avoid initiation of ERT in individuals without FD [4]. Our procedure resulted in separate criteria for male and female patients, and for patients with classical and non-classical FD. In general, initiation of ERT is recommended as soon as there are early signs of kidney, heart or brain involvement consistent with the diagnosis of FD. For male patients with classical FD treatment with ERT may even be considered before clinical signs or symptoms are evident, whereas the recommendation to start ERT in females with non-classical FD and early clinical signs is less strong. Our recommendation to initiate ERT when early clinical signs of FD occur is based on recent study reports showing that specific features of advanced disease, such as cardiac fibrosis or severe renal dysfunction, may compromise effectiveness of ERT. The presence of these features may point to irreversible organ damage [17,19,23]. Based upon these insights, early initiation of therapy is advocated. During the consensus procedure, however, it became clear that panellists differed in what they considered early. Some advocated starting treatment in classically affected males between the ages of 6 to 10 years. They argued that from a metabolic point of view ERT should be started as soon as possible. However, others expressed their preference to wait until the patient presents with signs or symptoms of organ involvement, especially since data showing benefit from starting ERT in the asymptomatic state are still lacking. Moreover, the paediatricians in the group in particular concurred in their view that early initiation of ERT in asymptomatic children in the context of a very slowly progressive disease is likely to interfere with normal childhood. It was finally agreed that initiation of ERT may be considered (Class IIB recommendation) in males with classical FD of 16 years or older even in the absence of any signs or symptoms. It was emphasised, however, that by far most males with classical disease will have signs or symptoms at the age of 16. With respect to commencing treatment in classically affected boys below the age of 16 years without clinical signs or symptoms, the consensus recommendations may be updated if new data supporting early treatment in children becomes available (www.clinicaltrials.gov, study number NCT00701415).

During the procedure, it was emphasised that having signs of organ involvement is an indication to start ERT only if organ damage is consistent with FD. All patients should therefore undergo extensive clinical and biochemical investigations, to map FD attributed organ damage and to identify other causes of kidney, heart or brain disease. This implies that in some cases a biopsy of an affected organ is necessary. For instance, an endomyocardial biopsy should be considered in patients with left ventricular hypertrophy as the sole presenting sign of organ involvement, especially when the patient has concomitant cardiovascular risk factors. Electron microscopic examination of biopsy specimen allows identification of other causes of organ damage. However, if storage characteristic of FD is seen, the cardiac hypertrophy is at least in part related to FD, and ERT is recommended.

Our recommendations assume that patients are receiving optimal supportive care. If ERT is started, angiotensin-converting enzyme (ACE) inhibitors or angiotensin II receptor blockers (ARB) should be started or continued according to the treatment guidelines for chronic kidney disease (KDIGO) [38] and cardiovascular disease (ESC) [39]. Antiplatelet therapy and statins are indicated if a patient has suffered a stroke or transient ischemic attack according to the AHA/ASA guidelines on prevention of recurrent strokes [40]. Pain medication may be needed if a patient has neuropathic pain.

The current recommendations are based on expert opinion and knowledge emerging from observational studies, since level A evidence (i.e. randomised controlled trials data) is scarce. Further studies are warranted to address effectiveness of ERT in patients with various degrees of kidney, heart or brain involvement. Panellists could, for example, not agree on a cut off value below which GFR indicates irreversible kidney disease. Some argued that treatment with ERT is not recommended for renal indication if the GFR is below 45 ml/min/1.73 m^2^ corrected for age (>40 years: −1 ml/min/1.73 m^2^/year), while others felt that there is still much to be gained when the GFR is below 45 ml/min/1.73 m^2^. So far, no studies have been reported that give an unequivocal answer to this dilemma. In addition, it was mentioned that the standard available follow-up procedures differ between centres. For example, specific cardiac magnetic resonance machines were used to detect cardiac fibrosis [37]. Whether the results can be extrapolated from one centre to others remains to be studied.

## Conclusion

Our recommendations have to be interpreted in the context of limited amount of evidence which implies that they need to be reconsidered when clinically relevant study results are published. Furthermore, the recommendations do not provide simple ‘go’ or a ‘no go’ rules; they should be used as a guidance for starting or stopping ERT in a FD patient while final decisions should be made on an individual basis taking into account FD features, concomitant diseases and personal preferences. The EFWG has the intention to update the recommendations on a regular basis and as such will act as an independent source of guidance for those involved in the care for patients with FD.

## Additional files

Additional file 1:
**Literature review results for the European Fabry Work Group (EFWG) consensus meeting on guidelines for initiation and cessation of enzyme replacement therapy (agalsidase alfa or agalsidase beta) in patients with Fabry disease.**
Additional file 2: Table S1. Statements on the initiation of ERT for which consensus was achieved. Additional file 3: Table S2. Statements on cessation of ERT or not starting ERT for which consensus was achieved. Additional file 4: Table S3. Statements for which no consensus was achieved.

## Abbreviations

FD: Fabry disease
GLA mutation: Mutation in the α-galactosidase A gene
AGAL-A: The lysosomal hydrolase α-galactosidase A
Gb3: Globotriaosylceramide
GVUS: Genetic variant of unknown significance
ERT: Enzyme replacement therapy
EFWG: European Fabry Working Group
FIN: Fabry International Network
FSIGN: Fabry Support and Information Group from the Netherlands
GFR: Glomerular filtration rate
LVH: Left ventricular hypertrophy
MWT: Maximal wall thickness
NYHA: New York Heart Association
CNS: Central nervous system
WMLs: White matter lesions
TIA: Transient ischemic attack
GI: Gastrointestinal
ACE: Angiotensin-converting enzyme
ARB: Angiotensin II receptor blocker
FU: Follow up
