# Supplementary material for: Recommendations for initiation and cessation of enzyme replacement therapy in patients with Fabry disease: the European Fabry Working Group consensus document
Source: Orphanet J Rare Dis. 2015 Mar 27;10:36. doi: 10.1186/s13023-015-0253-6 (PMC4383065; doi:10.1186/s13023-015-0253-6)
Supplement: Additional file 1: — Literature review results for the European Fabry Work Group (EFWG) consensus meeting on guidelines for initiation and cessation of enzyme replacement therapy (agalsidase alfa or agalsidase beta) in patients with Fabry disease. [file 13023_2015_253_MOESM1_ESM.doc]

**Literature review results for the European Fabry Work Group (EFWG) consensus meeting on guidelines for initiation and cessation of enzyme replacement therapy (agalsidase alfa or agalsidase beta) in patients with Fabry disease**

***Search strategy***

We collected all papers reporting on randomized controlled trials in order to give an overview of inclusion and exclusion criteria applied in these studies. To address the issue of limited effectiveness of ERT in patients with specific FD related features we searched for studies that report on renal or cardiac features potentially influencing the outcome of ERT (f.e. extensive cardiac fibrosis). For this purpose, we used the systematic search in Medline and Cochrane databases up until May 2013 performed by Rombach et al (1) supplemented with a Pubmed search for papers on FD published between May 2013 and March 2014. In addition, studies on the effects of ERT on the brain (TIA/strokes, white matter lesions) and acroparesthesias, and studies on the effectiveness of ERT in children were collected. Finally, two studies on effects of antibodies were included.

# *Literature review*

An overview of inclusion and exclusion criteria as applied in RCTs on the efficacy of ERT in FD is depicted in table 1. In general, study populations consisted mainly of males with classical FD. Since populations were selected for the specific purpose of these studies, the criteria have limited value to serve as a benchmark for start- and stop criteria.

The search for studies on renal or cardiac features potentially influencing the outcome of ERT revealed 10 and 8 articles respectively. The search revealed 6 studies on the effects of ERT on the brain (TIA/strokes, white matter lesions) and 2 on acroparesthesias. In addition, 6 studies on the effectiveness of ERT in children were found. Finally, 2 studies that have addressed the occurrence of antibodies against the infused enzymes were included. Study results are summarized in tables 2 to 5.

***Renal and cardiac features potentially influencing outcome of ERT***

***Renal features*:**

Table 2 summarizes nine studies that include renal features potentially influencing the outcome of ERT. In summary, four studies indicated that baseline proteinuria was associated with worse outcome of renal function: a more rapid decline in GFR is found in patients with a protein excretion of > 1g/24 hour (2-5). Glomerulosclerosis of > 50% was also a predictor of further decline in renal function (2). Progressive loss of renal function has been established to occur in patients with more advanced renal disease (5;6). In patients with hyperfiltration, the decline in eGFR is highest, perhaps reflecting normalization (4).

Overall complication rate is higher in patients with more advanced renal disease (7). Feriozzi also showed that hypertension was associated with poorer renal function (8). A higher dose of enzyme may lead to less rapid decline in renal function as shown in a clinical trial using weekly doses of agalsidase alfa instead of every other week (9), and worsening in eGFR and proteinuria in patients switching to lower dose due to enzyme shortage (10).

***Dialysis***

Pisani et al included dialysis patients in their study (11). They concluded that ERT is safe and effective, improving global QoL and possibly enhancing the progression of typical Fabry cardiomyopathy.

***Cardiac features:***

Table 3 summarizes 6 studies that addressed cardiac features potentially influencing cardiac responses. Patients with pre-existing left ventricular hypertrophy show in general a decline in LVmass, specifically during the first year of treatment (12). Longer term follow-up in a larger cohort showed stabilization in those without baseline LVH and improvement in those with LVH. Motwani et al showed that ERT improved LV morphology and function in patients with LVH — but there was no relationship between age, gender, FOS-MSSI or baseline ECG/TTE features and the response (13). ERT also normalised long QTc intervals, short PQ intervals and short P waves; and reduced disease burden (FOS-MSSI). Fibrosis in the heart has been shown as an important determinant of worse outcome (14;15): late enhancement on MRI in two or more segments is associated with lack of response to ERT (15). ERT in advanced Fabry disease seems of doubtful benefit (16). Despite ERT, clinically meaningful events including sudden cardiac death continue to develop in patients with advanced Fabry disease (10). In this study, increasing LE, cardiac mass and NYHA were associated with the occurrence of death or disease progression.

***Effects of ERT on the brain***

Table 4 summarizes studies on the effects of ERT on the brain. Development of white matter lesions has been described to occur or progress in both males and females after start of ERT (16;17). Jardim et al have made a similar observation, although they also described regression of some lesions (18). Occurrence of cerebrovascular events despite ERT is observed in several studies (3;16;17). In the phase IV agalsidase beta study, no TIA/strokes were reported in the treated group versus 2/31 new events in the placebo group. No statistically significant relationship with cardiovascular risk factors was found on the incidence of events (17). All patients in this study with risk factors received adequate support with antiplatelet therapy or lipid lowering drugs.

***Pain***

Acroparesthesias in FD patients have been reported to respond to carbamazepine (19). Also, beneficial effects of ERT have been observed in the pivotal trial on the effectiveness of ERT in FD (20). This RCT of 26 adult male patients used a decrease in pain as the primary outcome measure. The mean Brief Pain Inventory neuropathic pain severity score decreased from 6.2 to 4.3 in treated patients versus no significant change in the placebo group. However, these findings were not corroborated by another RCT of 58 patients in which statistically significant decreases in pain scores were observed in both the ERT and the placebo group (21). Since pain is a subjective outcome measure, and the course of pain in FD is variable and unpredictable, uncontrolled trials are unsuitable to estimate treatment effect.

***Children***

Treatment in children has so far mostly started in those with symptoms or signs, which were attributed to FD, such as pain, albuminuria and abdominal symptoms. Table 5 shows the studies in children with the inclusion criteria used for these studies. In general, ERT was safe and effective in reducing Gb3 and improving pain and well-being in several children. Long term effectiveness, e.g. the prevention of irreversible organ damage, has as yet not been shown. The FIELD study addresses pre-symptomatic start of therapy in classically affected boys, but so far no data have been published.

***Co-morbidities***

No studies have reported on benefits of ERT in patients with concomitant diseases.

No data are available on effects of ERT in case of co-existing vascular risk factors, except for a study by Feriozzi et al (8) who showed that hypertensive patients had a lower eGFR than normotensive patients at follow-up.

***Antibodies***

Several studies have addressed the occurrence of antibodies against the infused enzymes. These antibodies are usually neutralizing the activity of the enzyme and may alter the targeting. In patients with neutralizing antibodies, biochemical evidence of recurrence of storage is seen, by a rise in Gb3 or lysoGb3 in urine or plasma respectively (16). In line with this, Benichou observed recurrence of Gb3 accumulation in skin biopsies in patients with antibodies (22). So far, no study has shown an influence of antibodies on disease course or occurrence of complications. However, in the Netherlands, absence of a biochemical response in plasma lysoGb3 is considered as a criterion to interrupt treatment.

## Table 1: Overview of inclusion criteria of placebo-controlled studies and extension studies

| **Pivotal and**  **extension studies** | **Study design** | **Inclusion criteria** | **Exclusion criteria** | **Conclusion** |
| --- | --- | --- | --- | --- |
| **Schiffmann et al., 2001 (20)**  **Extension studies: Moore et al., 2001 (23)**  **Moore et al., 2002 (24)**  **Schiffmann et al., 2006 (25)** | Randomized, double-blind, placebo-controlled,  single-center trial Agalsidase-alfa | N=26 males, mean age 34 ± SD 2.26 (n=14) in placebo 34.4 ± SD 2.22 (n=12)  Inclusion criteria:   - Neuropathic pain - > 18 years - Enzymatically confirmed FD (residual activity <15%) | - Renal dialysis - Kidney Tx - Participating in other trials - Warfarin therapy | Intravenous infusions of recombinant a-galactosidase A are safe and have widespread therapeutic efficacy in FD |
| **Eng et al., 2001 (21)**  **Extension studies:**  **Thurberg et al., 2002 (26)**  **Thurberg et al., 2004 (27)** | Randomized, double-blind, placebo-controlled,  multicenter trial Agalsidase-beta | N=58; 56 males, 2 females, mean age 32 ± SD 9.4 (n=29) in placebo 28.4 ± SD 11.4 (n=29)  Inclusion criteria:   - Enzymatically confirmed FD (residual activity < 1.5 nmol/h/ml in plasma or < 4 nmol/h/ml in leukocytes) - > 16 years | - Renal dialysis - Kidney Tx - Serum creatinine concentration exceeded 2.2 mg per deciliter (194.5 μmol per liter) | Recombinant a-galactosidase A cleared microvascular endothelial deposits of globotriaosylceramide from the kidneys, heart, and skin in patients with FD |
| **Hajioff et al., 2003 (28)** | Randomized, placebo-controlled trial  Agalsidase-alfa | N=15 males, mean age 34.4 ± SD 3.8 (n=7), in placebo 36.9 ± SD 2.8 (n=8)  Inclusion criteria:   - Enzymatically or genetic ally confirmed FD - Between 25-49 years | NR | Agalsidase-alfa appears to  reverse the hearing deterioration in these patients |
| **Bierer et al., 2006 (29)** | Randomized, double-blind, placebo-controlled trial Agalsidase-alfa | N=15, mean age 32 ± (20-47)  Inclusion criteria:   - Enzymatically confirmed FD - Between 20-47 year | NR | Exercise tolerance increased in patients receiving ERT |
| **Banikazemi et al., 2007 (6)** | Randomized, double-blind,  placebo-controlled, multicenter trial Agalsidase-beta | N=82; 72 males, 10 females, mean age 46.9 ± SD 9.8 (n=51) in placebo group 44.3 ± SD 9.2 (n=31)  Inclusion criteria:   - no ERT history - > 16 years - Residual enzyme activity < 1.5 nmol/h/ml of plasma or < 4 nmol/h per mg of leukocyte protein - Serum creatinine >106 umol/L (> 1.2 mg/dL), < 265 umol/L (< 3.0mg/dL) OR - eGRF < 80 | - Renal dialysis - Renal Tx, - TIA, CVA, - Instable AP - MI within 3 months of trial entry - Clinically significant co-morbidities | Agalsidase-beta therapy slowed progression to the composite clinical outcome of renal, cardiac, and cerebrovascular complications and death compared with placebo in patients with advanced FD |
| **Hughes et al., 2008 (30)** | Randomized, double-blind, placebo-controlled trial Agalsidase-alfa | N= 15, males, mean age 37.1 ± SD 3.8 (n=7) in placebo 37.3 ± SD 2.9 (n=8)  Inclusion criteria:   - Diagnosis of FD confirmed by leukocyte AGAL-A activity and genotyping - Evidence of increased left ventricular mass by M-mode and two-dimensional echocardiography - > 18 year | - Presence of permanent cardiac pacemaker - Contraindication for cardiac biopsy | Treatment with agalsidase alfa resulted in regression of the hypertrophic  cardiomyopathy associated with FD |

## Table 2: Overview of studies on renal features that may influence the effectiveness of ERT

| **Author** | **Type of study and patient population**  **Purpose** | **Effect of ERT on kidney** | **Conclusion** |
| --- | --- | --- | --- |
| **Breunig et al., 2006 (7)** | Prospective, non-comparative, open label study  Patients:   - N=25; 19 males, 6 females - Mean age 39 ± 7.7 (GFR > 90), 43 ± 9.5 (GFR < 90) - Patients with GFR > 90 (n=9) AND patients with GFR < 90 (n=16)   Purpose: evaluate impact of ERT on heart and kidney function in patients with different stages of Fabry disease | Patients with GFR > 90  a. Reduced left ventricular posterior wall thickness (PWT)  b. Non-significant decrease of renal function -6.8 ml/min/1.73m^2^  c. No complications/events  Patients with GFR < 90  a. No reduction PWT  b. Significant decrease of renal function -5.7 ml/min/1.73m^2^  c. 12 events in 9 patients  Females(n=6): reduction of PWT and MDRD, events 2/6 | Patients with impaired renal function have a less favorable outcome and may develop cardiovascular and renal end points despite ERT |
| **Schiffmann et al., 2007 (9)** | Prospective, open label study  Patients:   - All patients with classic form of FD: no residual AGAL-A activity - N=41 males, 11 males selected - Mean age 44 ± SD 8.4 - eGFR decrease of > 5 ml/min/1.73m^2^ each year, but no ESRD   Purpose: evaluate effect of weekly dosing agalsidase alfa in pts with rapid decline in kidney function | Baseline: eGFR 77.8±30.4 ml/min per 1.73m^2^  Start weekly: eGFR 53.7±21.0 ml/min per 1.73m^2^  FU 2 years  EOW dosing: eGFR decline -8.0±2.8 ml/min per 1.73m^2^/yr Weekly dosing: eGFR decline -3.3±4.7 ml/min per 1.73m^2^/yr  9/11: positive eGFR slope (3/11) or slowing rate of decline (6/11)  2/11: failed to improve their slope | Weekly infusions of agalsidase alfa at a dosage of 0.2 mg/kg may be beneficial in the subgroup of patients who have FD and whose kidney function continues to decline after 2 to 4 yr or more of standard EOW dosing |
| **Banikazemi et al., 2007 (6)** | Randomized, double-blind, placebo-controlled trial  Patients:   - Enzymatically confirmed FD - N=82; 72 males, 10 females - Mean age 46.9 ± SD 9.8 and 44.3 ± SD 9.2 control group - Serum creatinine > 106 umol/L (> 1.2 mg/dL), < 265 umol/L (< 3.0mg/dL) OR - eGRF < 80   Purpose: evaluate long term impact of ERT on important clinical outcomes versus placebo | Patients with baseline GFR > 55 ml/min per 1.73m^2^ had stronger treatment effect than patients with GFRs of 55 ml/min per 1.73m^2^ or less  Patients with baseline serum creatinine levels of 132.6 umol/L or less had a greater treatment effect than those with baseline serum creatinine levels greater than 132.6 umol/l   No difference between patients with baseline proteinuria of 1 g/24h or less versus patients with proteinuria greater than 1 | ERT slowed progression to the composite clinical outcome of renal, cardiac, and cerebrovascular complications and death compared with placebo in patients with advanced FD.  The authors observed greater treatment effects in patients with less severe kidney  dysfunction at baseline |
| **Germain et al., 2007 (2)**  **Extension study of Eng et al., 2001 (21)** | Prospective, non-comparative, open-label study  Patients:   - Classical FD - N=58; 56 males, 2 females - mean age 32 ± 9.4 (n=29); placebo 28.4 ± 11.4 (n=29) - Serum creatinine < 2.2 mg/dL (194.5 mmol/L) - Extension follow-up additional 54 months   Purpose: evaluate long term impact of ERT | Histology: partial podocyte clearance of Gb3  Renal function stable in 52 patients (-0.4 ml/min/1.73m^2^/yr) and declines in 6 (range -3.5 to -12.1 ml/min/1.73m^2^/yr)  Baseline proteinuria > 1g/24h: decline in eGFR of -7.4 ml/min/ 1.73m^2^/yr; < 1g/24h: decline in eGFR - 1.0 ml/min/1.73m^2^/yr  Pretreatment glomerulosclerosis > 50% was associated with decline in eGFR of - 8.9 ml/min per 1.73m^2^/yr vs. 1.4 ml/min per 1.73m^2^/yr in pts with < 50% glomerulosclerosis | ERT stabilizes renal function in patients without renal involvement at baseline, maintains reduction of plasma Gb3, and sustains Gb3 clearance in capillary endothelial cells and multiple renal cell types. Proteinura and glomerulosclerosis have a negative impact on disease progression |
| **West et al., 2009 (4)** | Prospective cohort study  Patients:   - N=181 males - Mean age 34.2 ± SD 9.3   Purpose: effects of agalsidase alfa on kidney function in all adult male patients of earlier studies | GFR including patients with hyperfiltration: -4.8±10.6 ml/min per 1.73m^2^/yr  GFR excluding hyperfiltration: -2.9 ± 8.7 ml/min per 1.73m^2^/yr  GFR and proteinuria category (< 1 or ≥ 1 g/d) at baseline significantly predicted rate of decline of GFR | The response rate was better in patients with preserved kidney function at baseline  No effect on the proteinuria levels, but proteinuria negatively affects outcome |
| **Warnock et al., 2012 (5)** | Fabry Registry study  Patients:   - N= 213; 151 males, 62 females - Mean age m: 43, f: 38 - Sufficient measurements: serum creatinine, urinary protein:creatinine ratio (UP/Cr), eGFR values   Purpose: to identify determinants of renal disease progression in adults with Fabry disease during treatment with agalsidase beta | Males with:   1. UP/Cr ≥ 1 g/g: 4.5-fold higher risk of renal disease progression compared to UP/Cr < 0.3 g/g (p = 0.0006) 2. eGFR < 60 ml/min/1.73m^2^ at baseline had a 12-fold increase in the risk of being in eGFR slope -15.5 to -4.9 than males who had higher eGFR levels (p = 0.03).   Females with:   1. UP/Cr ≥ 1 g/g: 12-fold higher risk of renal disease progression compared to UP/Cr <0.3 g/g (p = 0.039) | Adults with Fabry disease are at risk for progressive loss of eGFR despite ERT, particularly if proteinuria is > 1 g/g  Men with urinary protein levels > 1 g/24h and lower baseline eGFR had a poorer response to ERT than those with lower urinary protein levels and better baseline eGFR |
| **Feriozzi et al., 2012 (8)** | Survey analysis  Patients:   - N=208; 134 males, 74 females - Mean age 38.4 ± SD 12.3 (m:34.5, f:45.6) - Treatment ≥ 5 years - Sufficient data on creatinine concentrations, proteinuria levels and eGFR at baseline   Purpose: to investigate the effectiveness of ERT with agalsidase alfa in treating Fabry nephropathy | eGFR: decline -2.2 ml/min per 1.73m^2^ in males (p < 0.01) and stabilization -0.7 ml/min per 1.73m^2^ in females, (p = 0.05)  Proteinuria: < 500 mg/24h slope –1.1 (-1.7; -0.6) ml/min/1.73m^2^ per year (n=120); 500-1000 mg/24h slope of –1.6 (-2.6; -0.7) ml/min/1.73m^2^ per year (n=28)  Arterial HT: hypertensive patients had a lower eGFR than normotensive patients at follow-up  ACEI/ABR: eGFR change -2.5 ±3.2 ml/min/1.73m^2^ with (n=58) and -1.1 ±2.5 ml/min/1.73m^2^ without ACEI/ABR (n=72) | Long-term ERT stabilizes the rate of nephropathy progression in females and is associated with a mild to moderate decline of renal function in males  Patients with 24-hour protein excretion >1 g/24h had poorer renal compared with patients with protein excretion of 500–1000 mg/24h or < 500 mg/24h |
| **Weidemann et al., 2013 (3)** | Prospective, comparative study  Patients:   - Genetically confirmed FD - N=40; 31 males, 9 females - Mean age 40 ± 9 (SD) - Treated > 5 years, or died - Matched with untreated FD patients   Purpose: effects of ERT on progression towards the end-points long-term survival and cause-specific death | - GRF decrease -2.3±4.6 ml/min (males 2.4±5.0 ml/min and females 1.9±3.3 ml/min  - In patients without proteinuria (<150 mg per day) (n=14) GFR remained stable over time  - Of 5 patient with proteinuria at baseline, 4 progressed to ESRD, and 1 had a decline in GFR from 48 to 27 ml/min | Progression of disease despite ERT  Proteinuria is associated with worse renal outcome |
| **Weidemann et al., 2014 (10)** | Prospective, observational cohort study  Patients:   - N=105; 62 males, 43 females - Mean age 45.3 ± SD 12.8 - Follow-up 39 ± SD 29 months   Purpose: assess clinical stability and safety during ERT dose reduction | eGFR decreased -3 ml/min/1.73m^2^ (p = 0.01) in the dose-reduction group, and the median albumin-creatinine ratio increased from 114 (0–606) mg/g to 216 (0–2062) mg/g (p = 0.03) in the switch group | Progression of albuminuria (nearly a 2.0-fold increase) and reduction of eGFR during ERT treatment switch to lower dose (agalsidase-beta, 0.3 or 0.5 mg/kg every other week) or switch to agalsidase-alfa (0.2 mg/kg) |

## Table 3: Overview of studies on cardiac features that may influence the effectiveness of ERT

| **Author** | **Type of study and patient population**  **Purpose** | **Effect of ERT on heart disease:** | | **Conclusion** |
| --- | --- | --- | --- | --- |
| **Beer et al., 2006 (14)** | Prospective case-control study  Patients:   - Genetically proven FD - N=35; 20 males, 15 females - Mean age 39 ± SD 10 - 12 months ERT (n=17)   Purpose: determine the impact of late enhancement indicative of fibrosis on regional myocardial function | Patients without fibrosis (n=9):   1. Reduction of LVM, 160 ± 23 g to 145 ± 27 g 2. Strain-rate improved   Patients with fibrosis (n=8):   1. No reduction of LVM (stable), 211 ±58 to 195 ±64 g 2. Stain rate improved on places without fibrosis | | Late enhancement may predict the effect of ERT on LVM and cardiac function |
| **Weidemann et al., 2009 (15)**  **Overlap in patiënts: Breunig et al., 2006 (7) Weidemann et al., 2003 (31)** | Prospective, non-comparative study  Patients:   - N=33; 27 males, 6 females - Mean age 42 ± SD 7 - 3 years ERT (None received ERT prior to study) - 14 patients had a history of HT - No fibrosis (n=12), mild fibrosis (n=11), or severe fibrosis (n=9)   Purpose: determine the impact of fibrosis on cardiac response | 1. Patients without fibrosis: effect PWT (p < 0.01), septum (p < 0.01), LVM (p < 0.01) 2. Patients with mild fibrosis: effect PWT (p < 0.21), septum (p = 0.20), LVM (p = 0.31) 3. Patients with severe fibrosis: effect PWT (p = 0.17), septum (p < 0.01), LVM (p = 0.24) | | Patients with myocardial fibrosis benefit less from ERT |
| **Kampmann et al., 2009 (12)** | Retrospective, pooled analysis, echocardiograms were assessed blinded  Patients:   - N= 45; 34 males, 11 females (96% white) - Mean age 39.8 ± SD 10.4 - 3 years ERT - Patients had LVH (n=14) or no LVH at baseline (n=31)   Purpose: determine the impact of LVH on cardiac response | Effect on LVM in males with LVH  a. 12 months ERT -11.8±7.5 g/m^2.7^ (n=6) b. 36 months ERT -3.2±8.2 g/m^2.7^ (n=7)  Effect on LVM in females with LVH  a. 12 months ERT -4.1±7.2 g/m^2.7^ (n=3) b. 36 months ERT -9.3±3.4 g/m^2.7^ (n=3) | Effect on LVM in males without LVH  a. 12 months ERT + 3.7±5.8 g/m^2.7^ (n=22) b. 36 months ERT +2.2±8.3 g/m^2.7^ (n=23)  Effect on LVM in females without LVH a. 12 months ERT + 3.0±5.7 g/m^2.7^ (n=6) b. 36 months ERT +0.9±5.9 g/m^2.7^ (n=3) | LVM did not differ between baseline and t = 36 months (effect on LVM in males with LVH, n=7, p=0.22; effect on LVM in males without LVH, n=23, p=0.26)  In females, the number was too small |
| **Motwani et al., 2012 (13)** | Retrospective analysis  Patients:   - Diagnosis confirmed by enzymatic or genetic analysis - N=66; 44 males, 22 female - Mean age 42 ± SD 14 - No ERT at baseline - Follow-up 3 years (range: 2-5 years) - 4 patients were excluded because of LBBB at baseline   Purpose: determine the impact of different disease parameters on cardiac response | Mean LVMI and MWT were reduced by ERT (LVMI: 116±28 vs. 113±26 g/m^2^, MWT: 14±6 vs. 13±5 mm; both p < 0.001)  a. pts with LVH at baseline (n=42) mean LVMI, MWT and LVEDD reduced by ERT (LVMI: 135±13 vs. 133±13 g/m^2^, MWT: 17±6 vs. 16±5 mm, LVEDD: 55±6 vs. 54±6 mm; all p < 0.05).  b. pts without LVH at baseline (n=24) : no change in mean LVMI, MWT or LVEDD (LVMI: 82±6 vs. 82±5 g/m^2^, MWT: 17±1 vs. 16±1 mm, LVEDD: 49±5 vs. 49±4 mm; all p > 0.05)  c. EF improvement in all (p < 0.05). Improved in the LVH sub-group (62±5 vs. 64±3%; p < 0.001) but no significant change in the non-LVH group (64±2 vs. 64±4%) | | ERT improved LV morphology and function in patients with LVH. No relationship between age, gender, FOS-MSSI or baseline ECG/TTE features and the response. ERT normalized long QTc intervals, short PQ intervals and short P waves |
| **Rombach et al., 2013 (16)** | Retrospective cohort study  Patients:   - N=75; 57 patients > 6 months follow-up; - 30 males, 27 females - Mean age LVM data; m: 37.1 ± SD 13.0 (n=27); f:46.6 ± SD 12.3 (n=26) - Comparison with untreated patients   Purpose: determine long term outcome of ERT | During ERT:  Males:   1. Cardiac mass increased +1.2 gram/m^2.7^, SE 0.3; p < 0.001) 2. LVH+: +1.5 ± 0.5, LVH-: +1.0 ± 0.3   Females:   1. Cardiac mass remained stable (-0.3 gram/m^2.7^ per year, SE 0.4; p=0.52 2. LVH+: -0.4 ± 0.6, LVH-: -0.3 ± 0.5 | | LVM remained stable in females, while LVM increased in males |
| **Weidemann et al., 2013 (3)** | Prospective, comparative study  Patients:   - Genetically confirmed FD - N=40; 31 males, 9 females - Mean age 40 ± 9 (SD) - Treated > 5 years, or died - Matched with untreated FD patients   Purpose: effects of ERT on progression towards the end-points long-term survival and cause-specific death | Myocardial LV fibrosis progression in males was 0.7±0.7%, in females 0.2±0.3%  Univariate analysis shows association with progression/death for: LE (5.17; 1.13–23.62); cardiac mass (per 10 g: 1.12; 1.03–1.23), GFR (per  ml/min: 0.98; 0.97–0.99); previous cerebrovascular accident (4.36; 1.14–16.64); NYHA) functional class (per class: 2.22; 0.93–5.26) and proteinuria (per 1 g: 1.31; 0.97–1.75). | | ERT has no effect on FD progression and death compared to no treatment in patients with advanced disease.  Increasing LE, cardiac mass and NYHA are associated with poor outcome. |

##

**Table 4**: Overview of studies on effectiveness of ERT on WML/strokes

| **Author** | **Type of study and patient population** |  | **Conclusion** |
| --- | --- | --- | --- |
| **Jardim et al., 2006 (18)**  **Extension study of Jardim et al, 2004 (32)** | Retrospective cohort study  Patients:   - Enzymatically proven Fabry disease - N=8; 7 males, 1 female (from 4 families) - Mean age: 32, range: 24-46 - 24 months of ERT (n=6) | Males: WML in 4/7 at baseline; 2/7: progression  M: WML: stable: 2/6, WML progression: 1/6, WML progression year 1, stable year 2: 1/6, WML regression:1/6  F: WML stable | A widespread pattern of silent WML in FD was seen. In two years, some WML appeared, and some disappeared. Effects of ERT cannot be determined |
| **Banikazemi et al., 2007 (6)** | Randomized, double-blind, placebo-controlled trial  Patients:   - Enzymatically confirmed FD - N=82; 72 males, 10 females - Mean age 46.9 ± SD 9.8   Purpose: evaluate long term impact of ERT on important clinical outcomes versus placebo | CNS events: TIA/stroke: 0 in agalsidase beta group (n=51) versus 2 in placebo group (n=31)  p = 0.14 | Beneficial effects of agalsidase beta to prevent complications; Power insufficient to draw conclusions about TIA/stroke |
| **Buechner et al., 2008 (17)** | Retrospective cohort study  Patients:   - N=43; 25 males, 18 females - Mean age: m: 41.9 ± SD 10.8 (n=25), f: 52.5 ± SD 17.5 (n=18) - Agalsidase alfa (n=10), agalsidase beta (n=14) and no ERT (n=19) | Males: no ERT WML progression: 1/3, ERT WML progression: 3/10  Females: no ERT WML progression: 3/6, ERT WML progression: 2/5  Males: no ERT new CV events: 1/3, ERT new CV events: 5/17  Females: no ERT new CV events: 4/9, ERT new CV events: 2/6 | Progression of WMLs and cerebrovascular events despite ERT is common  No statistically significant relationship with cardiovascular risk factors and outcome |
| **Weidemann et al., 2013 (3)** | Prospective, comparative study  Patients:   - Genetically confirmed FD - N=40; 31 males, 9 females - Mean age 40 ± 9 (SD) - Treated > 5 years, or died - Matched with untreated FD patients   Purpose: effects of ERT on progression towards the end-points long-term survival and cause-specific death | Strokes occur in treated patients (4/40 compared to 10/40 in untreated patients)  Univariate analysis shows association with progression/death for: previous cerebrovascular accident (4.36; 1.14–16.64); | ERT has no effect on FD progression and death compared to no treatment in patients with advanced disease |
| **Rombach et al., 2013 (16)** | Retrospective cohort study  Patients:   - N=75; 57 patients had at least 6 months follow-up; 30 males, 27 females - Mean age WML data; m: 37.3 ± SD 12.9 (n=25), f: 46.6 ± SD 12.8 (n=25) - All were symptomatic (CKD, LVH, WML) | Males: WML progression: 12/25 (48%)  Females: WML progression: 7/25 (28%) | Long term ERT does not prevent disease progression, but the risk of developing a first or second complication declines with increasing treatment duration. Several treated patients had progression of WML's. Power insufficient to draw conclusions about TIA/stroke |

**Table 5**: Overview of studies on the effectiveness of ERT in children

| **Author** | **Type of study and patient population** | **Effect of ERT** | **Conclusion** |
| --- | --- | --- | --- |
| **Ries et al., 2006 (33)** | Prospective, non-comparative, open-label, multicenter study  Patients:   - Enzymatically (boys) or genetically (girls) confirmed FD - N=24; 19 boys, 5 girls - Mean age: 11.8, range 6-18 - 6 months ERT - No history of ERT | Mean GFR, cardiac structure, and function were normal at baseline no change over 26 weeks  Boys:  - Reduction in plasma Gb3 and urine Gb3  - Heart rate variability improved | ERT reduced plasma Gb3 levels in patients with elevated baseline levels and reduced the use of pain medications |
| **Ramaswami et al. 2007 (34)** | Prospective  Patients:   - Enzymatically (boys) or genetically (girls) confirmed FD - N=13; 9 boys, 4 girls - Mean age 11.0, range 2-18 - 24 weeks ERT - Adequate general health | - BPI scores decreased  - Pain-related QoL scores decreased  - Plasma Gb3, urinary Gb3 decreased | ERT reduced plasma and urine sediment Gb3 levels in boys and resulted in improvement in pain in the entire study population |
| **Wraith et al., 2008 (35)** | Prospective, non-comparative, open-label study  Patients:   - Enzymatically (boys) or genetically (girls) confirmed FD - N=16; 14 boys and 2 girls - Mean age: 12.1 ± SD 2.5, range 8-15 - 48 weeks ERT - ≥ Tanner Stage III - ≥ 1 of following: pain crises, chronic pain; urine albumin level > 30 mg/dL; eGFR < 80 ml/min; post-prandial abdominal pain, nausea, or vomiting; autonomic neuropathy (hypohidrosis, impaired pupillary constriction, reduced tear production); low BMI; abbreviated P-R interval - Skin biopsies (n=12) | - Skin Gb3 deposits were cleared or reduced (n=13, week 24)  - Plasma Gb3 decreased from 15.9 ug/ml to 6.3 ug/mL  - Urinary protein excretion was 118±55 mg/m^2^/24h at baseline (n=15), 77±29 at week 24 (n=15), and 95±35 at week 48 (n=13)  - No variation of serum creatinine  - GFR was 126±29 ml/min/1.73m^2^ at baseline (n=16), 128±29 at week 24 (n=16), and 125±26 at week 48 (n=15)  - Good general health increased from 69% to 77% | ERT safely and effectively reduced the Gb3 accumulation in dermal endothelium already evident in children with FD. Early intervention may prevent irreversible end-organ damage from chronic Gb3 deposition |
| **Schiffmann et al., 2010 (36)** | Prospective, non-comparative, open-label extension study  Patients:   - Enzymatically (boys) or genetically (girls) confirmed FD - N=17; 16 boys, 1 girl - Mean age: NR range 7-18 - 6 months ERT (n=17) + 3.5 years extension (n=10, 9 boys) | a. Plasma Gb3 levels (boys) declined  b. Mean urine Gb3 levels (boys) were reduced to normal range (p < 0.05)  c. No reduction of LVM, stable  d. Kidney function remained stable  e. Gastrointestinal symptoms improved  f. Boys reported less pain  - Boys: IgG antibodies (n=1) | ERT well tolerated and associated  with improvement of FD related features |
| **Ramaswami et al., 2011 (37)** | Retrospective chart review, non-comparative study  Patients:   - Enzymatically (boys) or genetically (girls) confirmed FD - N=8; 7 boys, 1 girl - Mean age: 5.0 ± SD 1.6, range 6-18 - Start ERT age ≤ 7 years - Symptomatic (pain) | - Mean GFR was normal, remained normal  - 5/6 LVMi was above the 75^th^ percentile of age-matched children (baseline), 2/6 after ERT  - Boys: IgG antibodies (n=1) | Long-term observation will be needed to determine whether early initiation of ERT will prevent major organ dysfunction in these patients |
| **Borgwardt et al., 2013 (38)** | Retrospective, ERT follow-up maximum 8 years  Patients:   - Genetically confirmed FD - N=10; 6 boys, 4 girls - Mean age: NR, range 9-16 - Ophthalmological, echocardiographic abnormalities and hypohidrosis | - No reduction of LVM  - Majority of boys had improved sweating capacity  - Plasma and urine Gb3 decreased to normal range after 2-33 months  - 8/10 reported a decrease in acroparaesthesia | The authors argue that early initiation of ERT may be considered because there is a good response on chronic pain |

**Abbreviations**

ACE = angiotensin I-converting enzyme inhibitors

AGAL-A = the lysosomal hydrolase α-galactosidase A

AP = angina pectoris

ARB = angiotensin receptor blockers

BMI = body mass index

BPI = brief pain inventory

CKD = chronic kidney disease

CRI = chronic renal insufficiency: creatinine >1.5 mg/dl

CV = cardiovascular

ECG = electrocardiography

eGFR = estimated glomerular filtration rate

EOW = every other week

ERT = enzyme replacement therapy

ESRD = End stage renal disease

f = female

FD = Fabry disease

FOS-MSSI = Fabry Outcome Survey - Mainz Severity Score Index

Gb3 = globotriaosylceramide

HT=hypertension,

Ig = immunoglobulin

LE = late enhancement

LVEDD = left ventricular end-diastolic diameter

LVH = left ventricular hypertrophy

LVM = left ventricular mass

LVMi = left ventricular mass index

m = male

MWT = maximal wall thickness

NR = not reported

PWT = left ventricular posterior wall thickness

QoL = quality of life

TTE = transthoracic echocardiogram

Tx = transplantation

Up/Cr = urinary protein:creatinine ratio

WML = cerebral white matter lesions

Reference List

(1) Rombach SM, Smid BE, Linthorst GE, Dijkgraaf MG, Hollak CEM. Natural course of Fabry disease and the effectiveness of enzyme replacement therapy: a systematic review and meta-analysis: effectiveness of ERT in different disease stages. J Inherit Metab Dis 2014; 37(3):341-352.

(2) Germain DP, Waldek S, Banikazemi M, Bushinsky DA, Charrow J, Desnick RJ et al. Sustained, long-term renal stabilization after 54 months of agalsidase beta therapy in patients with Fabry disease. J Am Soc Nephrol 2007; 18:1547-1557.

(3) Weidemann F, Niemann M, Störk S, Breunig F, Beer M, Sommer C et al. Long-term outcome of enzyme-replacement therapy in advanced Fabry disease: evidence for disease progression towards serious complications. J Intern Med 2013; 274(4):331-341.

(4) West M, Nicholls K, Mehta A, Clarke JT, Steiner RD, Beck M et al. Agalsidase alfa and kidney dysfunction in Fabry disease. J Am Soc Nephrol 2009; 20(5):1132-1139.

(5) Warnock DG, Ortiz A, Mauer M, Linthorst GE, Oliveira JP, Serra AL et al. Renal outcomes of agalsidase beta treatment for Fabry disease: role of proteinuria and timing of treatment initiation. Nephrol Dial Transplant 2012; 27(3):1042-1049.

(6) Banikazemi M, Bultas J, Waldek S, Wilcox WR, Whitley CB, McDonald M et al. Agalsidase-beta therapy for advanced Fabry disease: a randomized trial. Ann Intern Med 2007; 146(2):77-86.

(7) Breunig F, Weidemann F, Strotmann JM, Knoll A, Wanner C. Clinical benefit of enzyme replacement therapy in Fabry disease. Kidney Int 2006; 69(7):1216-1221.

(8) Feriozzi S, Torras J, Cybulla M, Nicholls K, Sunder-Plassmann G, West M. The effectiveness of long-term agalsidase alfa therapy in the treatment of Fabry nephropathy. Clin J Am Soc Nephrol 2012; 7(1):60-69.

(9) Schiffmann R, Askari H, Timmons M, Robinson C, Benko W, Brady RO et al. Weekly enzyme replacement therapy may slow decline of renal function in patients with Fabry disease who are on long-term biweekly dosing. J Am Soc Nephrol 2007; 18(5):1576-1583.

(10) Weidemann F, Kramer J, Duning T, Lenders M, Canaan-Kühl S, Krebs A et al. Patients with Fabry disease after enzyme replacement therapy dose reduction versus treatment switch. J Am Soc Nephrol 2014; 18(5):1576-1583.

(11) Pisani A, Spinelli L, Sabbatini M, Andreucci MV, Procaccini D, Abbaterusso C et al. Enzyme replacement therapy in Fabry disease patients undergoing dialysis: Effects on quality of life and organ involvement. Am J Kidney Dis 2005; 46(1):120-127.

(12) Kampmann C, Linhart A, Devereux RB, Schiffman R. Effect of agalsidase alfa replacement therapy on Fabry disease-related hypertrophic cardiomyopathy: a 12- to 36-month, retrospective, blinded echocardiographic pooled analysis. Clin Ther 2009; 31(9):1966-1976.

(13) Motwani M, Banypersad S, Woolfson P, Waldek S. Enzyme replacement therapy improves cardiac features and severity of Fabry disease. Mol Genet Metab 2012; 107(1-2):197-202.

(14) Beer M, Weidemann F, Breunig F, Knoll A, Koeppe S, Machann W et al. Impact of enzyme replacement therapy on cardiac morphology and function and late enhancement in Fabry's cardiomyopathy. Am J Cardiol 2006; 97(10):1515-1518.

(15) Weidemann F, Niemann M, Breunig F, Herrmann S, Beer M, Störk S et al. Long-term effects of enzyme replacement therapy on fabry cardiomyopathy: evidence for a better outcome with early treatment. Circulation 2009; 119(4):524-529.

(16) Rombach SM, Smid BE, Bouwman MG, Linthorst GE, Dijkgraaf MG, Hollak CE. Long term enzyme replacement therapy for Fabry disease: effectiveness on kidney, heart and brain. Orphanet J Rare Dis 2013; 8:47.

(17) Buechner S, Moretti M, Burlina A, Cei G, Manara R, Ricci R et al. Central nervous system involvement in Anderson-Fabry disease: A clinical and MRI retrospective study. J Neurol Neurosurg Psychiatry 2008; 79(11):1249-1254.

(18) Jardim LB, Aesse F, Vedolin L, Pitta-Pinheiro C, Marconato J, Burin MG et al. White matter lesions in Fabry disease before and after enzyme replacement therapy: a 2-year follow-up. Arq Neuropsiquiatr 2006; 64(3B):711-717.

(19) Filling-Katz MR, Merrick HF, Fink JK, Miles RB, Sokol J, Barton NW. Carbamazepine in Fabry's disease: effective analgesia with dose-dependent exacerbation of autonomic dysfunction. Neurology 1989; 39(4):598-600.

(20) Schiffmann R, Kopp JB, Austin HAI, Sabnis S, Moore DF, Weibel T et al. Enzyme replacement therapy in Fabry disease: a randomized controlled trial. JAMA 2001; 285:2743-2749.

(21) Eng CM, Guffon N, Wilcox WR, Germain DP, Lee P, Waldek S et al. Safety and efficacy of recombinant human alpha-galactosidase a replacement therapy in Fabry's disease. N Engl J Med 2001; 345(1):9-16.

(22) Benichou B, Goyal S, Sung C, Norfleet AM, O'Brien F. A retrospective analysis of the potential impact of IgG antibodies to agalsidase beta on efficacy during enzyme replacement therapy for Fabry disease. Mol Genet Metab 2009; 96(1):4-12.

(23) Moore DF, Scott LTC, Gladwin MT, Altarescu G, Kaneski C, Suzuki K et al. Regional cerebral hyperperfusion and nitric oxide pathway dysregulation in Fabry disease: Reversal by enzyme replacement therapy. Circulation 2001; 104(13):1506-1512.

(24) Moore DF, Altarescu G, Ling GSF, Jeffries N, Frei KP, Weibel T et al. Elevated cerebral blood flow velocities in Fabry disease with reversal after enzyme replacement. Stroke 2002; 33:525-531.

(25) Schiffmann R, Ries M, Timmons M, Flaherty JT, Brady RO. Long-term therapy with agalsidase alfa for Fabry disease: safety and effects on renal function in a home infusion setting. Nephrol Dial Transplant 2006; 21(2):345-354.

(26) Thurberg BL, Rennke H, Colvin RB, Dikman S, Gordon RE, Collins AB et al. Globotriaosylceramide accumulation in the Fabry kidney is cleared from multiple cell types after enzyme replacement therapy. Kidney Int 2002; 62(6):1933-1946.

(27) Thurberg BL, Randolph Byers H, Granter SR, Phelps RG, Gordon RE, O'Callaghan M. Monitoring the 3-year efficacy of enzyme replacement therapy in Fabry disease by repeated skin biopsies. J Invest Dermatol 2004; 122(4):900-908.

(28) Hajioff D, Enever Y, Quiney R, Zuckerman J, Mackermot K, Mehta A. Hearing loss in Fabry disease: the effect of agalsidase alfa replacement therapy. J Inherit Metab Dis 2003; 26(8):787-794.

(29) Bierer G, Balfe D, Wilcox WR, Mosenifar Z. Improvement in serial cardiopulmonary exercise testing following enzyme replacement therapy in Fabry disease. J Inherit Metab Dis 2006; 29(4):572-579.

(30) Hughes DA, Elliot PM, Shah J, Zuckerman J, Coghlan G, Brookes J et al. Effects of enzyme replacement therapy on the cardiomyopathy of Anderson-Fabry disease: a randomised, double-blind, placebo-controlled clinical trial of agalsidase alfa. Heart 2008; 94(2):153-158.

(31) Weidemann F, Breunig F, Beer M, Sandstede J, Turschner O, Voelker W et al. Improvement of cardiac function during enzyme replacement therapy in patients with Fabry disease: a prospective strain rate imaging study. Circulation 2003; 108(11):1299-1301.

(32) Jardim L, Vedolin L, Schwartz IV, Burin MG, Cecchin C, Kalakun L et al. CNS involvement in Fabry disease: clinical and imaging studies before and after 12 months of enzyme replacement therapy. J Inherit Metab Dis 2004; 27(2):229-240.

(33) Ries M, Clarke JTR, Whybra C, Timmons M, Robinson C, Schlaggar BL et al. Enzyme-replacement therapy with agalsidase alfa in children with Fabry disease. Pediatrics 2006; 118:924-932.

(34) Ramaswami U, Wendt S, Pintos-Morell G, Parini R, Whybra C, Leon Leal JA et al. Enzyme replacement therapy with agalsidase alfa in children with Fabry disease. Acta Paediatr 2007; 96(1):122-127.

(35) Wraith JE, Tylki-Szymanska A, Guffon N, Lien YH, Tsimaratos M, Vellodi A et al. Safety and efficacy of enzyme replacement therapy with agalsidase beta: an international, open-label study in pediatric patients with Fabry disease. J Pediatr 2008; 152(4):563-570.

(36) Schiffmann R, Martin RA, Reimschisel T, Johnson K, Castaneda V, Lien YH et al. Four-year prospective clinical trial of agalsidase alfa in children with Fabry disease. J Pediatr 2010; 156(5):832-837.

(37) Ramaswami U, Parini R, Kampmann C, Beck M. Safety of agalsidase alfa in patients with Fabry disease under 7 years. Acta Paediatr 2011; 100(4):605-611.

(38) Borgwardt L, Feldt-Rasmussen U, Rasmussen AK, Ballegaard M, Meldgaard Lund A. Fabry disease in children: agalsidase-beta enzyme replacement therapy. Clin Genet 2013; 83(5):432-438.
