# Supplementary material for: Recommendations for initiation and cessation of enzyme replacement therapy in patients with Fabry disease: the European Fabry Working Group consensus document
Source: Orphanet J Rare Dis. 2015 Mar 27;10:36. doi: 10.1186/s13023-015-0253-6 (PMC4383065; doi:10.1186/s13023-015-0253-6)
Supplement: Additional file 2: Table S1. — Statements on the initiation of ERT for which consensus was achieved. [file 13023_2015_253_MOESM2_ESM.doc]

*Appendix table 1: Statements on the initiation of ERT for which consensus was achieved*

| **Statement**  **number** | **Statement** | **Consensus** | **Class** |
| --- | --- | --- | --- |
| 3 | Treatment with ERT is recommended in young male patients at the age 0-5 years with classical FD without any symptoms/clinical signs of organ involvement at the time this patient is being investigated | Negative | I |
| 6 | Treatment with ERT may be considered in a newly diagnosed young male patients of 16 years or older with classical FD without any symptoms/clinical signs of organ involvement at the time this patient is being investigated | Positive | IIB |
| 7 | Treatment with ERT is recommended as soon as possible after a diagnosis of FD is made in a non-classical male patients without any symptoms/clinical signs of organ involvement | Negative | I |
| 8 | Treatment with ERT is recommended as soon as possible after a diagnosis of FD is made in a female patients without any symptoms/clinical signs of organ involvement | Negative | I |
| 9a | Treatment with ERT is recommended in males with classical FD as soon as there are early signs of organ involvement (kidney, heart and/or brain signs) consistent with FD (early signs not specified here) | Positive | I |
| 9b | Treatment with ERT is recommended in males with non-classical FD as soon as there are early signs of organ involvement (kidney, heart and/or brain signs) consistent with FD and not fully explained by other pathology (early signs not specified here) | Positive | I |
| 10a | Treatment with ERT is recommended in females with classical FD as soon as there are early signs of organ involvement (kidney, heart and/or brain signs) consistent with FD and not fully explained by other pathology (early signs not specified here) | Positive | I |
| 10b | Treatment with ERT may be considered in females with non-classical FD as soon as there are early signs of organ involvement (kidney, heart and/or brain signs) consistent with FD and not fully explained by other pathology (early signs not specified here) | Positive | IIB |
| 12 | Treatment with ERT is recommended in patients with FD with cardiac hypertrophy (>12 mm) without (or only minimal signs of) fibrosis consistent with FD and not fully explained by other pathology | Positive | I |
| 14 | Treatment with ERT is recommended in patients with FD with signs of cardiac rhythm disturbances (sinus bradycardia, AF, repolarization disorders) consistent with FD and not fully explained by other pathology | Positive | I |
| 15a | Treatment with ERT is recommended in male patients with FD with early signs of renal disease consistent with FD and not fully explained by other pathology | Positive | I |
| 15b | Treatment with ERT may be considered in female patients with FD with early signs of renal disease consistent with FD and not fully explained by other pathology | Positive | IIB |
| 17a | Treatment with ERT is recommended in male patients with FD with microalbuminuria (according to international guidelines of kidney disease, KDIGO criteria) consistent with FD and not fully explained by other pathology | Positive | I |
| 17b | Treatment with ERT may be considered in female patients with FD with microalbuminuria (according to international guidelines of kidney disease, KDIGO criteria) consistent with FD and not fully explained by other pathology | Positive | IIB |
| 18 | Treatment with ERT is recommended in male patients with FD with proteinuria (according to international guidelines of kidney disease, KDIGO criteria) consistent with FD and not fully explained by other pathology | Positive | I |
| 20a | Treatment with ERT is recommended in male patients with classical FD who have renal insufficiency (GFR 60-90 ml/min/1.73m^2^ corrected for age (> 40 years: -1 ml/min/1.73m^2^/year)) | Positive | I |
| 20b | Treatment with ERT should be considered in male patients with non-classical FD who have renal insufficiency (GFR 60-90 ml/min/1.73m^2^ corrected for age (> 40 years: -1 ml/min/1.73m^2^/year)) | Positive | IIA |
| 20c | Treatment with ERT should be considered in female patients with classical FD who have renal insufficiency (GFR 60-90 ml/min/1.73m^2^ corrected for age (> 40 years: -1 ml/min/1.73m^2^/year)) | Positive | IIA |
| 21a | Treatment with ERT may be considered in male patients with classical FD who have renal insufficiency (GFR 45-60 ml/min/1.73m^2^ corrected for age (> 40 years: -1 ml/min/1.73m^2^/year)) | Positive | IIB |
| 21b | Same for patients with non-classical FD and females | Positive | IIB |
| 25 | Treatment with ERT may be considered in patients with FD who have WMLs | Positive | IIB |
| 26 | Treatment with ERT should be considered in patients with FD who have had a TIA/stroke | Positive | IIA |
| 29 | Treatment with ERT may be considered in patients with FD who have hearing loss, corrected for age | Positive | IIB |
| 30a | Treatment with ERT should be considered in patients with FD with GI symptoms | Positive | IIA |
| 30b | Treatment with ERT should be considered in patients with FD < 16 years with GI symptoms | Positive | IIA |
| 30c | Treatment with ERT may be considered in patients with FD > 16 years with GI symptoms | Positive | IIB |
| 31 | Treatment with ERT should be considered in patients with FD who have acroparesthesias | Positive | IIA |
| 32a | Treatment with ERT is recommended in patients with FD who have acroparesthesias even if they are completely controlled (not interfering with daily activities) with carbamazepine (or other medication) | Negative | I |
| 32b | Treatment with ERT may be considered in patients with FD who have acroparesthesias even if they are completely controlled (not interfering with daily activities) with carbamazepine (or other medication) | Positive | IIB |
