# Supplementary material for: Recommendations for initiation and cessation of enzyme replacement therapy in patients with Fabry disease: the European Fabry Working Group consensus document
Source: Orphanet J Rare Dis. 2015 Mar 27;10:36. doi: 10.1186/s13023-015-0253-6 (PMC4383065; doi:10.1186/s13023-015-0253-6)
Supplement: Additional file 3: Table S2. — Statements on cessation of ERT or not starting ERT for which consensus was achieved. [file 13023_2015_253_MOESM3_ESM.doc]

*Appendix table 2: Statements on cessation of ERT or not starting ERT for which consensus was achieved*

| **Statement**  **number** | **Statement** | **Consensus** | **Class** |
| --- | --- | --- | --- |
| 13 | Treatment with ERT is not recommended for cardiac indication in patients with FD and advanced cardiac disease with extensive fibrosis consistent with FD and not fully explained by other pathology | Positive | I |
| 21c | Renal insufficiency (GFR < 45 ml/min/1.73m^2^ corrected for age (> 40 years: -1 ml/min/1.73m^2^/year)) should not preclude treatment with ERT in male patients with classical FD | Positive | I |
| 21d | Same for patients with non-classical FD and females | Positive | I |
| 23 | Treatment with ERT should not be withheld in patients with FD on dialysis who can receive a renal transplant | Positive | I |
| 24 | Treatment with ERT should not be withheld in patients with FD on dialysis who are not eligible for a renal transplant | Positive | I |
| 42a | It is recommended to stop treatment with ERT in male patients with classical FD when lack of response for 1 year when the sole indication for ERT is neuropathic pain while receiving maximum supportive care | Negative | I |
| 42b | It may be considered to stop treatment with ERT in female patients with classical FD when lack of response for 1 year when the sole indication for ERT is neuropathic pain while receiving maximum supportive care | Positive | IIB |
| 50a | Treatment with ERT should not be withheld in patients with FD and cognitive decline of any cause (provided the patient has the capacity to consent to treatment) | Positive | I |
| 50b | Stopping ERT may be considered in patients with severe cognitive decline of any cause | Positive | IIB |
| 52 | Stopping ERT may be considered in patients with end stage FD or other comorbidities with a life expectancy of < 1 year | Positive | IIB |
| 53 | Stopping ERT is recommended in patients with FD who are non-compliant > 50% of infusions | Positive | I |
| 54 | Stopping ERT is recommended in patients with FD who fail to show up regularly (according to local guidelines) at FU visits | Positive | I |
| 55 | ERT should be stopped in patients with FD at patient request | Positive | I |
| 56 | ERT should be stopped in patients with FD who have persistent life threatening or severe infusion reactions that do not respond to prophylaxis, e.g. anaphylaxis | Positive | I |
| 58 | Stopping ERT should be considered in patients with FD and: end stage renal disease (provided that the patient is not eligible for transplantation) AND end stage heart failure | Positive | IIA |
