# Supplementary material for: Recommendations for initiation and cessation of enzyme replacement therapy in patients with Fabry disease: the European Fabry Working Group consensus document
Source: Orphanet J Rare Dis. 2015 Mar 27;10:36. doi: 10.1186/s13023-015-0253-6 (PMC4383065; doi:10.1186/s13023-015-0253-6)
Supplement: Additional file 4: Table S3. — Statements for which no consensus was achieved. [file 13023_2015_253_MOESM4_ESM.doc]

*Appendix table 3: Statements for which no consensus was achieved*

| **Statement**  **number** | **Statement** |
| --- | --- |
| 2 | Treatment with ERT should be initiated in young male patients at any age range with classical FD without any symptoms/clinical signs of organ involvement at the time this patient is being investigated |
| 4 | Treatment with ERT may be considered in young male patients at the age 6-10 years with classical FD without any symptoms/clinical signs of organ involvement at the time this patient is being investigated |
| 5 | Treatment with ERT may be considered in a newly diagnosed young male patients at the age 11-15 years with classical FD without any symptoms/clinical signs of organ involvement at the time this patient is being investigated |
| 11 | Treatment with ERT may be considered in patients with FD with early signs of cardiac disease (unspecified) consistent with FD and not fully explained by other pathology |
| 16 | Treatment with ERT is recommended in patients with FD with podocyte storage only (as shown on kidney biopsy) |
| 21e | Treatment with ERT is not recommended for renal indication in male patients with classical FD who have renal insufficiency (GFR < 45 ml/min/1.73m^2^ corrected for age (> 40 years: -1 ml/min/1.73m^2^/year)) |
| 21f | Treatment with ERT may be considered in male patients with classical FD who have renal insufficiency (GFR < 45 ml/min/1.73m^2^ corrected for age (> 40 years: -1 ml/min/1.73m^2^/year)) |
